# The Epidemiology of Sports-Related Head Injury and Concussion in Water Polo

**DOI:** 10.3389/fneur.2016.00098

**Published:** 2016-06-24

**Authors:** Robert S. Blumenfeld, Jessica C. Winsell, James W. Hicks, Steven L. Small

**Affiliations:** ^1^Department of Psychology and Sociology, California State Polytechnic University, Pomona, CA, USA; ^2^Department of Neurology, University of California Irvine, Irvine, CA, USA; ^3^Department of Ecology and Evolutionary Biology, University of California Irvine, Irvine, CA, USA; ^4^Brain Circuits Laboratory, Biological Sciences III, University of California Irvine, Irvine, CA, USA

**Keywords:** concussion, brain injury, sports-related head injury, epidemiology, survey, mild TBI, contact sports

## Abstract

**Purpose:**

Water polo is a sport with a high degree of physicality and aggressive play. Unlike most contact sports, epidemiological data on the incidence or prevalence of head trauma in water polo have not been gathered, reported, or made publicly available. The purpose of this study was to begin a systematic characterization of the risks of head impact and concussion in men and women who play water polo at various levels.

**Design:**

We sent an electronic survey to the 44,000+ members of USA Water Polo, asking questions about concussions, head impacts, and symptoms commonly associated with prior concussion. From over 1500 complete responses, we report summary information on the prevalence of concussions and major head impacts in water polo.

**Results:**

We found that 36% of respondents report sustaining a concussion while playing water polo, with an average of two concussions reported. The prevalence and number of concussions reported varied across positions, levels, and gender. Most strikingly, we found that goalies are at significantly higher risk for concussion, report a significantly more concussions, and appear to experience a qualitatively different type of head impact compared to other positions. Additionally, we found that competition level, gender, and field position are robust predictors of concussion risk.

**Conclusion:**

Our findings demonstrate that concussions are not uncommon in water polo players. We conclude that there is need for systematic concussion reporting in water polo and suggest that understanding the risk factors of concussion in water polo will require fully considering differences in the head impact exposure between different field positions, competition levels, sexes, and differences in exposure between competition and practice.

## Introduction

In recent years, there has been growing awareness of the risks of head injury, concussion, and brain injury in sports (1–3). This increased societal awareness has driven a broad spectrum of research investigating the nature and risk factors for head injuries in contact sports across the lifespan, across genders, and across levels of play. Understanding the nature, incidence, and prevalence of sports-related concussion is a major focus of both clinical and basic research.

Two decades ago, the American Academy of Neurology defined concussion as “an alteration of mental status due to a biomechanical force affecting the brain” (4). More recently, arguing that concussion and mild traumatic brain injury (mTBI) are interchangeable terms, the American Congress of Rehabilitation Medicine defined these more precisely as “traumatically induced physiological disruption of brain function resulting from the head being struck or striking an object or the brain undergoing an acceleration and deceleration movement, as manifested by at least one of the following: any period of loss of consciousness up to 30 min; posttraumatic amnesia not exceeding 24 h; any period of confusion or disorientation; transient neurological abnormalities, including focal signs, seizures, and intracranial lesions not requiring surgery; and a [neurological evaluation ranging from] confusion to normal consciousness on examination within 30 min after presentation” (5). Long-term functional consequences of concussions, frequently described as *post-concussive syndrome (PCS)* (6), may include compromised cognitive, sensory-motor, and psychosocial outcomes. The risk factors that lead to long-term and persistent symptoms, so called *refractory PCS*, are complex and controversial, and although not fully understood, include age of injury and a number of psychosocial factors, among others. Multiple concussions as well as repeated sub-concussive blows to the head and impacts at particular head locations are increasingly implicated in determining long-term risk.

To assess the risk of head injury and concussion in a particular sport, it is crucial to have available epidemiological data on concussions, sub-concussive blows, and associated risk factors within that sport. At the American college level, the incidence of concussion in football, hockey, lacrosse, and soccer are reported annually. The publication of this information is made possible because there are standardized reporting procedures in place for individual schools, regional conferences, and the national governance organizations. Football, hockey, lacrosse, and soccer are popular sports known for their physicality, and indeed, head injuries in these sports alone accounted for 64% of all reported sports-related concussions in the past year (7). Of these, hockey, lacrosse, and soccer are popular for both men and women, and it is known that head injuries in these sports do not affect men and women to the same degree or with the same consequences (7–9). At the college level, approximately 24–25% of male athletes and 21–29% of female athletes playing these sports report experiencing at least one concussion (10). Studies examining the nature and prevalence of head injuries in these sports are certainly important. At the same time, there are other sports with growing popularity, such as water polo, where concussion and head impact data are not being systematically reported.

Water polo is highly physical and is increasingly popular for both men and women. It is well-known to higher level (e.g., scholastic, collegiate) players that there is a considerable risk of (unintended) physical injury from elbows, kicks, high velocity hits by the ball, and other consequences of the game. In addition, as in most other physical sports, there is also a degree of intentional competitive physicality. In water polo, at all levels of the sport, there is no standardized set of procedures for reporting concussion, and notably water polo is one of the only NCAA sanctioned sports in which an injury database is not available (7). Moreover, to the best of our knowledge, there are no published data reporting incidence or prevalence of concussion or head impacts in water polo at any level of the sport.

Here, we provide the first report of epidemiological data on the nature of concussions and sub-concussive blows to the head in water polo. We conducted a large internet-based survey of current, former, and associate members of USA Water Polo, asking respondents about their level of experience playing water polo, and their history of serious blows to the head, including identified concussions, while playing. We received complete responses from over 1500 members (3.38% of members), covering a broad range of ages, experience levels, and positions played across both genders.

From these data, we sought to address the following set of questions: Are concussions occurring in the sport of water polo? Are the risks of concussion similar at all levels and positions in the sport of water polo? Are there particular subgroups of players who are at higher risk because of their age, position, level of play, and/or gender? Further, we sought to quantify the frequency with which players receive serious blows to the head, in and out of the game, and the nature of head impacts experienced by players in different positions.

## Materials and Methods

### Population

We received a total of 2060 unique responses to our survey. Five hundred forty one respondents consented to participate in the survey but left their surveys incomplete, and consequently this report is based on responses for 1519 unique respondents. All respondents were members, former members, or associates of USA Water Polo. Of the 1470 respondents that reported gender, 40% (602 respondents) were women, and 60% (895 respondents) were men. Counts of respondents for High School, College, and Masters Club levels of play, for each position are summarized in Table 1. See Table S1 in Supplementary Material for the number of respondents across all levels of play.

**Table 1:** **Number of respondents, lifetime concussion prevalence, and average number of concussions**.

	Number of respondents	Lifetime concussion prevalence	Average number of concussions^a^
High school	606	193 (31.8%)	1.6 ± 0.1
College	199	102 (51.2%)	2.3 ± 0.1
Masters club	390	168 (43.1%)	2.5 ± 0.2
Attacker (A)	320	116 (36.25%)	2.0 ± 0.2
Utility (U)	397	104 (26.2%)	2.0 ± 0.1
2-m Offense (2mO)	227	83 (36.5%)	2.0 ± 0.1
2-m Defense (2mD)	273	108 (39.56%)	2.1 ± 0.1
Goalie (G)	255	120 (47.1%)	2.5 ± 0.2
Female	602	257 (43.5%)	2.1 ± 0.1
Male	889	266 (30.8%)	2.2. ± 0.1

*^a^Average number of concussions in respondents reporting 1 or more concussions*.

### Design

A web link to the survey, along with a brief description, was sent by email to all 40,284 current, former, and affiliate members of USA Water Polo on April 13, 2015 as part of a regular USA Water Polo Newsletter. Clicking on the link navigated respondents to the first page of the survey containing a study information sheet. After confirming by checkbox that they had read the study information sheet and were in agreement to participate in the survey, respondents entered the survey questionnaire page.

### Survey Tool

The survey consisted of 21 questions grouped into four general categories.

#### Demographic Information

We gathered information on age, gender, years of experience, levels of the sport played (e.g., high school, college, masters) and whether the respondent remains an active player. *Sub-concussive head impacts*: we asked questions about the frequency of serious blows to the head and how these blows occurred. We asked how often they were hit by the ball or by other players; whether these hits occurred during games or during practices; and where on their head (location of impacts) they were hit. Third, we gathered data on respondents’ personal histories with concussion in water polo.

#### Concussion

We first provided a simple descriptive definition of concussion signs and symptoms, as “a blow to the head followed by a variety of symptoms that may include any of the following: headache, dizziness, loss of balance, blurred vision, ‘seeing stars,’ feeling in a fog or slowed down, memory problems, poor concentration, nausea, or throwing up. Getting ‘knocked out’ or being unconscious does NOT always occur in concussion” (10). We then asked if the respondent had ever had a concussion during water polo play. If so, we asked about the cumulative number of concussions experienced during play and whether the respondent had one or more seasons where he or she experienced multiple concussions. Finally, we gathered data on symptoms commonly associated with PCS.

#### Symptoms

We asked respondents whether they experience headaches, problems sleeping, or irritability on a regular basis. The full questionnaire is presented in Table S2 in Supplementary Material.

### Data Processing

Before reporting on the data, several decisions were required to systematize analysis and reporting of special cases. First, not every respondent answered every question. To account for this, null responses were not counted, and all reported percentages were adjusted for the number of responses available for each variable. Second, for several questions in the survey, we requested respondents to type in a specific number [e.g., “Even if you do not think it resulted in a concussion, how many serious blows to the head have you had while playing water polo? (Please provide a specific number”)]. However, responses to these questions permitted answers of any format. For respondents who provided a range of values (e.g., “5–10”), we substituted the mean value of the range. For respondents who answered in an open-ended manner (e.g., “more than three times” or “less than a dozen”), we added or subtracted one to the number given (e.g., “4” or “11”). In total, only 16 responses for the “number of concussion” were estimated (mean estimated number of concussion = 2.9 ± 0.43; median = 2.5). In a small number of cases, respondents entered a non-quantitative value (e.g., “a few” or “countless”), and these responses were omitted from further analysis.

### Dependent Measures

Two measures served as our major dependent variables. Our primary dependent variable was whether an individual respondent reported sustaining one or more concussions (see Table S2 in Supplementary material, question 11). From this we calculated projected lifetime prevalence of concussion as the percentage of respondents in a particular group of interest who report sustaining at least one concussion over their playing career. A secondary dependent measure – for those individuals who reported at least one concussion – was the mean number of reported concussions over their playing lifetime.

### Analysis

All statistical analysis was performed in Python (11) using several standard packages. To calculate highest competition level, we first ranked the levels of play in the following order: 1: age group club (e.g., “12 and under,” “14 and under”); 2: high school, 3: college, 4: masters club; 5: olympic; 6: professional. Maximum level was then determined as the highest level/rank that a given player participated in from the lowest level (1; age group club) to the highest (6; professional). To examine head impact locations, we calculated an “impact location score” for each position based on the answers to four questions about location of impact. This score was calculated as follows: for each impact location (e.g., “back of the head,” “front of the head”), the number of responses in each response bin (i.e., 0: “none of the time,” 1: “some of the time,” 2: “most of the time,” 3: “all of the time”) were counted and then divided by the total number of responses. This provides impact location percentages. Each percentage was then multiplied by a weighting factor (0 for “none of the time” through 3 for “all of the time”), and the weighted percentages were then summed to provide a weighted impact score for each location. The weighted impact location scores across all locations were evaluated for each field position.

For the final quantification, removed outliers by omitting responses that were higher than 3 SD from the mean (those who reported 12 or more concussions).

We conducted statistical modeling of concussion and symptom reporting to characterize the factors influencing these reports. Two models were tested for reports of concussion. In one logistic regression model, the report of at least one concussion was treated as a binary dependent variable, with gender, position (goalie or not goalie), and highest competition level assigned as predictors. Second, we performed multiple linear regression, using the same predictors, but now counting the number of reported concussions as a dependent variable. For analysis of symptoms, the number of symptoms reported was treated as the dependent variable and gender, position (goalie or not goalie), highest competition level, and a report of one or more concussions were assigned as predictors. For each of these analyses, highest competition level was treated as an ordinal variable (from Age Group Club, High School, College, Masters Club, Olympic, Professional). Field position was treated as a dichotomous variable, comparing goalies to non-goalies. In the results, the following acronyms for field position are used: Attackers (A), Utility (U), 2-Meter Offense (2mO), 2-Meter Defense (2mD), and Goalie (G).

## Results

### Concussions

**Figure 1:**
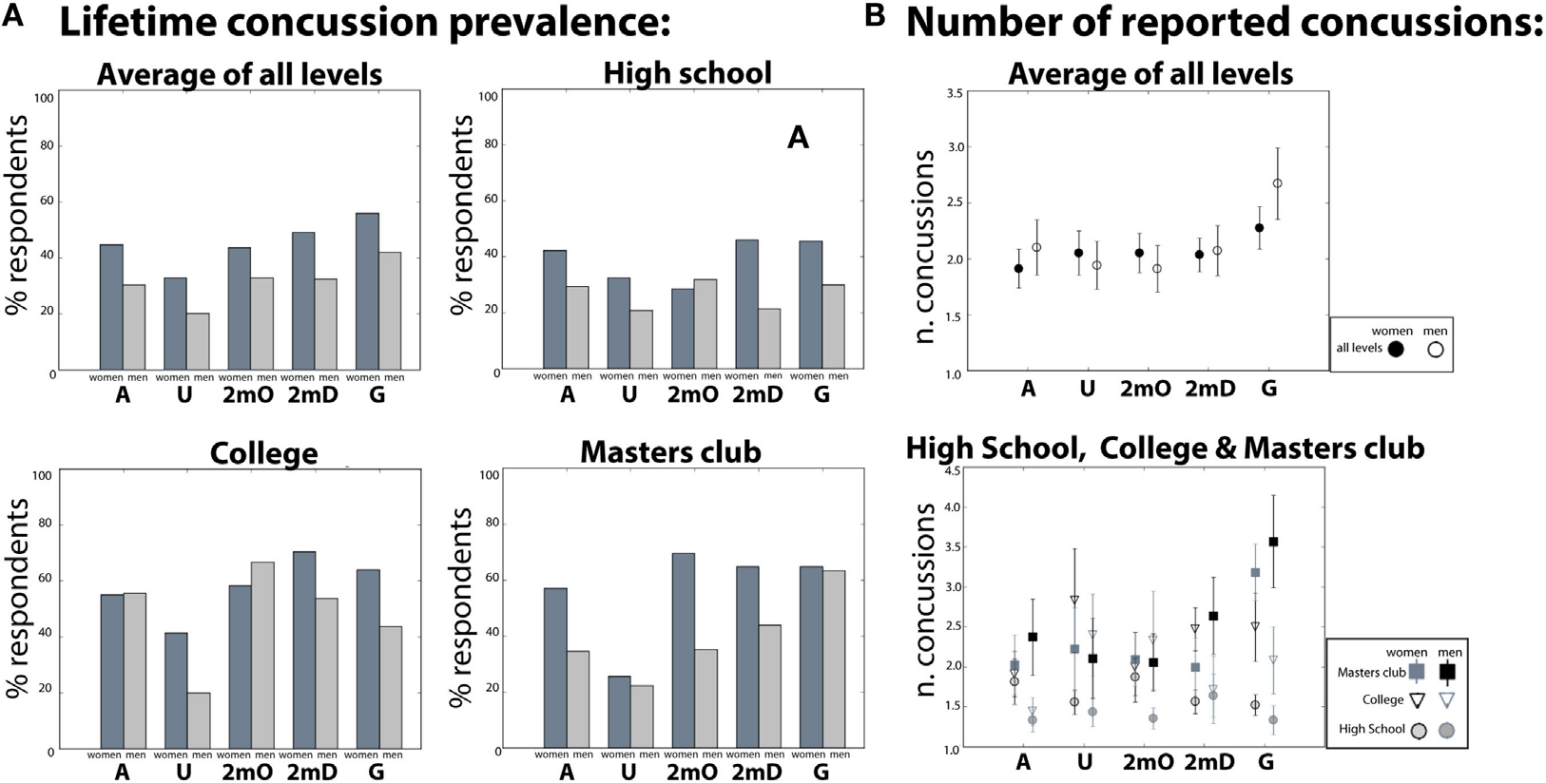
**(A)** Lifetime concussion prevalence and the **(B)** number of concussions by field position reported in high school, college, masters club and averaged across all levels.

Table 1 and Figure 1A present projected lifetime prevalence and average number of reported concussions broken down by field position, gender, and level of play. 534 (36.0%) of all 1485 respondents reported sustaining at least one concussion during water polo play. In this group of 534, respondents reported sustaining an average of 2.14, SE (±) = 0.07 concussions. Examining projected lifetime prevalence according to gender, we found that 266 males (30.8%) and 257 female (43.5%) reported sustaining at least one concussion.^1^ Men reporting concussion sustained an average of 2.20 ± 0.12 concussions. Women reporting concussion sustained an average of 2.06 ± 0.08 concussions. Examining concussion reporting by field position, we found that 120 or 47% of goalies reported sustaining at least one concussion. This projected lifetime prevalence was higher than all other field positions [*χ*^2^(1, *N* = 1485) = 20.5, *p* = 5.9 × 10^−6^]. This group of 120 goalies reported an average of 2.49 ± 0.18 concussions. Next, we examined concussion reporting as a function of level of play. For these analyses, we grouped respondents according to the highest competition level reported (see Materials and Methods). Of the 606 respondents whose highest competition level was in High School, 193 (31.8%) report at least 1 concussion and an average of 1.58 ± 0.07 concussions. Of the 199 respondents whose highest competition level was in College, 102 (51.3%) report at least 1 concussion with an average 2.29 ± 0.14 concussions. Of the 390 respondents whose highest competition level was at the masters level (Master’s Club), 168 (43.1%) report at least 1 concussion, with a mean number of concussions of 2.52 ± 0.15. Notably, the percentage of College players sustaining at least one concussion was significantly greater than that of Masters’ Club, despite the fact that those whose highest level of play was at the Masters’ Club level had significantly more years of play (i.e., exposure) than those whose highest competition level was at the College level [*χ*^2^(2, *N* = 589) = 5.43, *p* = 0.019], for both players with and without concussion. Note also that not all respondents with Master’s Club level as their highest competition level necessarily played at the College level. There were 304 respondents who played at both College and Master’s Club levels, and reported Master’s Club as their maximum level. Of this group, 138 (45.4%) report at least one concussion, which is a lower prevalence than that of maximum level College players. See Table S2 in Supplementary Material for reporting on the additional levels of play.

Using logistic regression, we formally tested whether individuals’ reporting of one or more concussions could be predicted by respondents’ gender, field position, and highest competition level. Specifically, we tested whether respondents’ gender, whether or not they played the goalie position, and the highest competition level (see Materials and Methods) predicted whether or not respondents reported at least one concussion. Gender [odds-ratio = 0.91, *t*(639) = −3.05, *p* = 0.002, Goalie status odds-ratio = 1.12, *t*(639) = −2.53, *p* = 0.01], and highest competition level [odds-ratio = 1.05, *t*(639) = −3.95, *p* = 0.0001] all significantly predicted concussion and together explained a significant amount of variance [*R*^2^ = 0.05, *X^2^*(2, 639) = 21.78, *p* = 3.1 × 10^−6^, see Table S3A in Supplementary Material for details]. These data indicate that women players, goalies, and playing at progressively higher levels of the sport increase the likelihood of concussion.

Next, using multiple linear regression, we formally tested whether the number of concussions could be predicted by respondents’ gender, field position, and/or highest competition level. Goalie status [β = 0.58, *t*(639) = 3.829, *p* = 1.4 × 10^−4^] and maximum level [β = 0.41, *t*(639) = 8.25, *p* < 8.8 × 10^−16^] significantly predicted the number of concussions but gender [β = −0.133, *t*(639) = −1.09, *p* = 0.42] did not (See Table S2B in Supplementary Material). Further planned comparisons showed that maximum level college women reported significantly more concussions than men [*t*(109) = 2.55, *p* = 0.01 see Figure 1].

### Sub-Concussive Head Impacts

**Figure 2:**
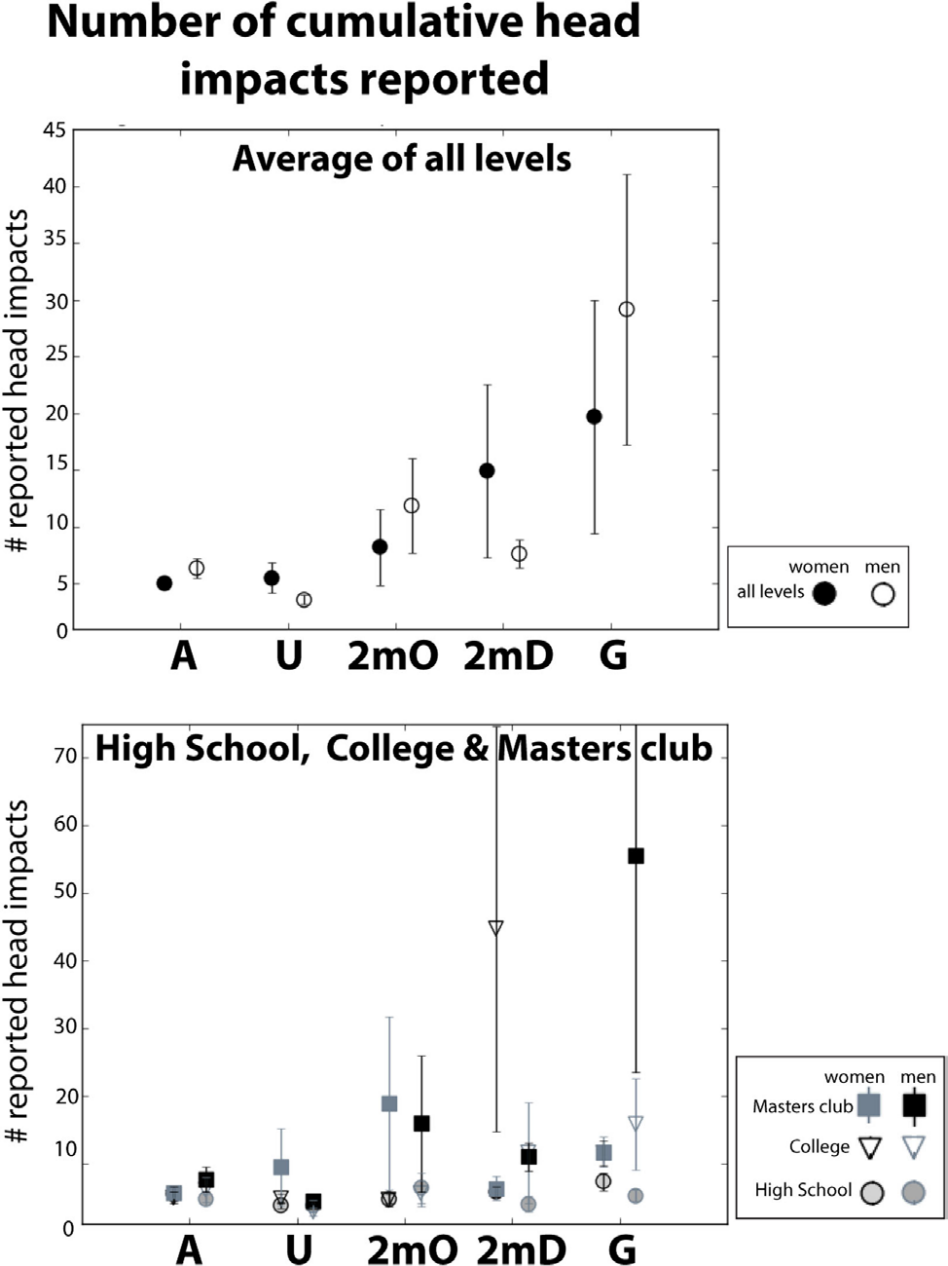
**Lifetime cumulative number of serious head impacts by field position reported in high school, college, masters club, and averaged across all levels**. Error bars reflect the SE of the mean.

We included several items in our survey examining respondents’ experience with head impacts and sub-concussive blows. In particular, we asked respondents several questions pertaining to how, when, and how frequently they are/were hit in the head during water polo. We first report our findings on the self-reported cumulative number of serious head impacts stemming from the question: “*Even if you don’t think it resulted in a concussion, how many serious blows to the head have you had while playing water polo?*” The mean cumulative number of head impacts reported across all respondents was 10.67 ± 1.61. This is further broken down by gender, position, and maximum level in Figure 2 (see also Table S3 in Supplementary Material). As can be seen Figure 2, cumulative blows to the head increase with maximum level [*F*(598) = 2.34, *p* = 0.04]. In addition, a correlation between the number of cumulative head impacts and years of play indicated that the number of reported head impacts increases with years played [*r*(597) = 0.08, *p* = 0.04].

**Figure 3:**
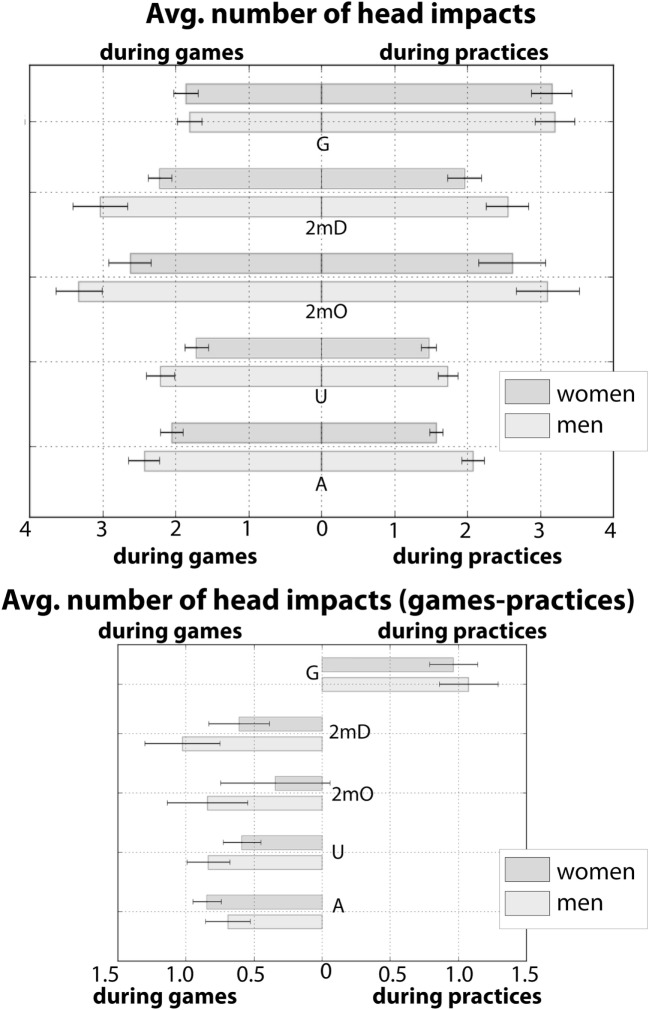
**Average number of reported head impacts during a “typical game” and a “typical practice” for men and women**. In the top bar graph, position is presented on the *y*-axis. The leftward pointing bars left indicated the average number of blows reported during a “typical game” and rightward pointing bars indicate indicated the average number of blows reported during a “typical practice.” The bottom bar plot is the subtraction (game-practice) of the leftward and rightward pointing bars from the top plot. All positions except for goalies report more head impacts during games. Error bars reflect the SE of the mean.

Respondents also reported the average number of “serious blows to the head” in a given practice and a given game (See Table S2 in Supplementary Material, Questions: 7–8). We report this in Figure 3. The mean number of “serious blows to the head” during a “typical” practice is 1.85 ± 0.08 and 2.27 ± 0.07 during a “typical” game across all positions. As can be seen in Figure 3, when broken down by gender and position, all positions except goalies report more blows during games compared to practice. Goalies report a mean of 2.81 ± 0.19 blows during practice and 1.84 ± 0.12 during games.

**Figure 4:**
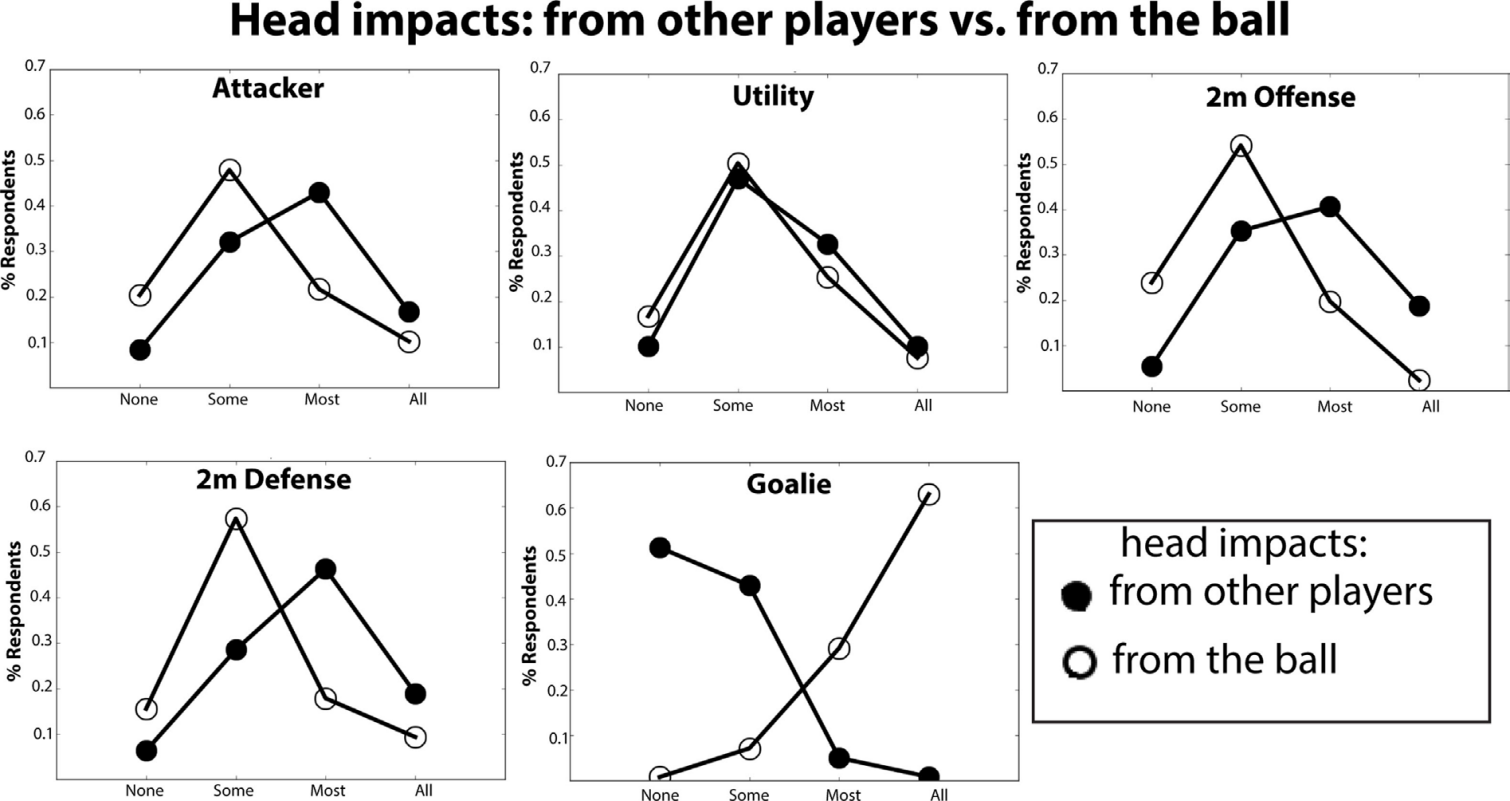
**Histogram of reported prevalence of head impacts caused by other players vs**. **the ball**. On the *y*-axis is plotted the % of respondents that indicated that their heads’ were struck: none, some, most, or all of time by other players (closed circles) or by the ball (open circle).

We next report on responses from the two survey questions on the frequency of head impacts caused by the ball compared to head impacts from other players (see Table S2 in Supplementary Material Questions: 9E–F). Figure 4 (open circles) shows the distribution of % responses (from 0: never – 3: all the time) for “*head impacts from the ball*” across each position, and Figure 4 (closed circles) shows the distribution of % responses (from 0: never – 3: all the time) for “*head impacts from other players*” across each position. As can be seen from these plots, in all positions except for goalies, the frequency distributions for “hits by other players” are centered rightward – indicating a higher frequency of head impacts from other players in these positions. In the frequency distributions for “head impacts by the ball” all positions except for the goalie position are centered leftward – indicating a lower frequency of head impacts from the ball. By contrast, in goalies this response distribution is centered on the extreme right and the response distribution for “head impacts from other players” responses is centered on the extreme left. Indeed, over 60% of goalies report that when their head is impacted, it is “always” from the ball and 50% of goalies report that their head has been hit both other players “none” of the time.

**Figure 5:**
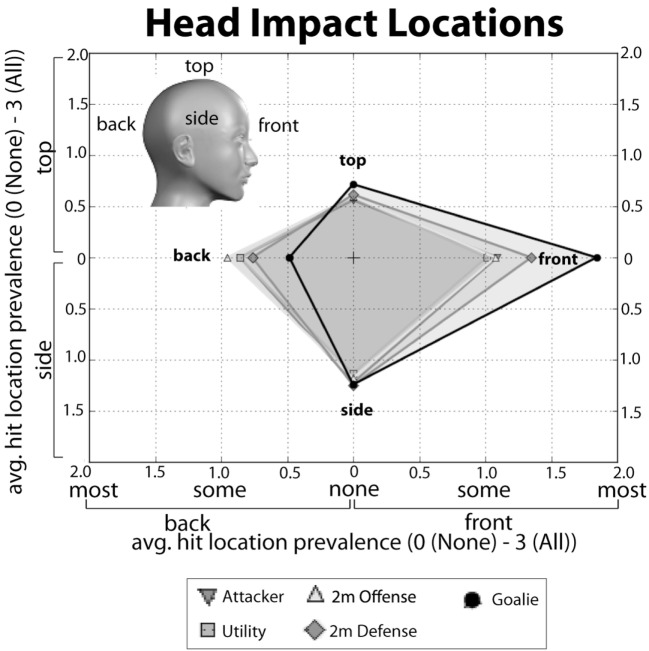
**Head impact location “fingerprints” for each position**. The average prevalence score (“0-None of the time” to “3-All of the time”) reported for impacts to the back (−*x*), top (+*y*), front (+*x*), or side (−*y*) of the head. Each score is plotted along the axes given the parentheses above. The fingerprint for each position is the polygon created by connected each impact score for that position along the *x* and *y* axes.

We additionally asked respondents about where on their heads they are typically hit during water polo (Table S2 in Supplementary Material, Questions: 9A–D). Specifically, we asked respondents to report how frequently (from 0: “Never” to 3: “Always”) their heads are being impacted on the back of their heads, on the sides of their heads, the front of their heads, and on the top their heads. We calculated a weighted “impact location score” based on the percentage of responses for each head impact location and for each field position. These data are presented in Figure 5. Each position is represented as polygon centered on the origin. For each polygon, the values on the +*x*, −*x*, +*y*, and −*y* axes represent the impact location score for the back, front, top, and side of the head, respectively. The unique shapes of these polygons provide a head impact location “*fingerprint*” for each position. The fingerprints show that the front and the side of the head are the most frequently reported head impact locations. Defense and Goalie report the “front of the head” as the most frequent location and Attackers, Centers and Utility players report the “side of the head” more frequently. Although each fingerprint is distinct, there is considerable spatial overlap between the Attackers, Utility and 2 m positions. This indicates that these positions have similar distributions of impact location scores. In contrast, the Goalie fingerprint appears more skewed along the +*x* direction compared to all other positions, indicating that goalies report that they sustain hits to the front of the head with much greater frequency compared to the other hit locations. Overall, the impact location fingerprints reported here are quite consistent with what might be expected based on the role and location on the field for each position.

### Symptoms Associated with Post-Concussive Syndrome

In our third set of analyses, we examined the frequency at which respondents indicated that they experienced three symptoms associated with PCS (see Table S2 in Supplementary Material, Question 19: “headaches,” “problems sleeping,” “irritability”). The mean number of reported symptoms was 0.39 ± 0.03 (median = 0) for men and 0.85 ± 0.04 (median 1) for women across all respondents. The number of symptoms significantly differed between those who reported at least one concussion (0.96 ± 0.05) and those who reported no concussions (0.36 ± 0.02) [*χ*^2^(3, *N* = 1491) = 197.07, *p* = 1.81 × 10^−42^]. These data, further broken down by gender are presented in Table S4A in Supplementary Material. We also examined the frequency of reported symptoms as a function of the number of reported concussions. These data are presented in Table S4B in Supplementary Material and demonstrate that the mean number of reported symptoms increases with the number of concussions reported (from the one concussion to two, from two to three, from three to five, and from five and above). Using logistic regression, we tested whether the number of symptoms reported would serve as a reliable predictor of concussion prevalence. We found that number of symptoms reported was a significant positive predictor of concussion prevalence [odds-ratio = 1.15, *t*(638) = 6.0, *p* = 3.2 × 10^−10^ see Table S2C in Supplementary Material]. Analysis of the mean data (Table S4 in Supplementary Material) revealed that among individuals with at least one concussion, women report more symptoms than men [*t*(522) = 5.87, *p* = 7.73 × 10^−9^].

### Removal of Outliers and Examining Effects of Selection Bias

Selection and self-reporting bias is often a concern in survey research. In this study, one possible concern is that a select set of outlier respondents might have over-reported their number of concussions and head impacts. Thus, as mentioned in the Methods, one way we attempted to address this concern is by removing outliers prior to all statistical analyses (See Materials and Methods).

The definition of concussion that we used, following McCrea and colleagues (12), provided a simple description of the signs and symptoms of concussion. It did not constrain concussion reporting to certain fixed time periods in the past and also it allowed respondents to include as concussions self-reported head impacts that were not clinically diagnosed as concussions. Given this, there is the potential for a complex set of factors related to differences in respondents’ age (e.g., generational differences and memory differences) to skew potential results. For instance, older individuals, who may have experienced concussions more than a decade prior, might systematically underreport their number of concussions because they may not have remembered every instance of concussion. Further, older individuals may underreport because of generational differences in how they subjectively define concussion compared to younger individuals.^2^ Because of these concerns, we explored age differences in our sample. Notably, the majority (80%) of respondents in our sample were under 30. We compared the projected concussion prevalence in our “younger” (≤30) and “older” (>30) and incidence rates. The prevalence in older (42.51%) and younger individuals (34.37%) was found to be different [χ*^2^*(1, *N* = 1485) = 8.43, *p* < 0.001]. Similarly, the number of concussions reported between older and younger individuals were found to be significantly different [*t*(521) = 3.68, *p* < 0.001]. Thus there are age clear differences. However, most importantly, comparing the prevalence of the younger individuals (34.37%) to the prevalence overall (35.95%), we found no significant difference [χ^2^(1, *N* = 1185) = 1.57, *p* > 0.05]. Thus, we found no evidence that differences in reporting by individuals over 30 produce a bias that skewed the overall numbers that are reported for projected prevalence.

In an additional set of analyses, we compared the reporting of head impacts (see Table S2 in Supplementary Material, question 16) in respondents who report suffering at least one concussion compared to those who did not report concussion. If those reporting concussions provided biased accounts, it is likely that they would exaggerate the number of blows to the head they experienced in games, practices, or cumulatively compared to those respondents that did not report a concussion. Conversely, if bias were not the driving factor guiding responses for respondents reporting concussions, then we might expect the number of blows in respondents without concussion to be slightly reduced but overall to have a similar pattern of blows in games vs. practices. As can be seen in Table S1 in Supplementary Material, respondents who report suffering at least one concussion do report an overall higher number of blows during games, practices and cumulatively. However, the proportion of hits in games vs. practices was not significantly different between these two groups (all *t*’s < 1.60, all *p*’s > 0.05).

## Discussion

Water polo is a highly physical contact sport that is growing in popularity across the United States. Importantly, the incidence and prevalence of concussion in the sport are not known. Here, we sought to address this gap in knowledge by gathering epidemiological data on concussions in water polo using an internet-based survey. In this report, we present descriptive statistics and summaries on concussion and head impacts from 1491 completed survey records. As can be seen from our data, concussions are reported across all positions and all levels and in both men and women. These data represent a necessary and important first step in understanding the prevalence of concussion in water polo.

As this is the first systematic survey of concussions in water polo, we note several important limitations of our study that must be considered and addressed in follow-up work. First, although we received responses from approximately 2060 individuals, this represents fewer than 4% of the estimated water polo players in the US, and thus, we cannot claim that the data reported here are representative of all water polo players. Second, given that our survey was internet-based, self-report, self-selecting, and relied on participants’ memory for events, and to some extent, on respondents’ subjective definition of concussion, we cannot rule out the possibility of systematic bias or the over-reporting of concussions. However, though the projected lifetime prevalence of concussion that we observed (35%) does not, *ipso facto*, suggest a systematic bias or skew in selection. Further, although our sample is relatively small, the gender balance, and the distribution of positions, and participation across the levels of the sport are all reflective of the greater water polo population. That is, this sample’s demographics mirror that of typical team rosters. Further, to reduce potential bias, all cases of outliers (concussions >12) were removed before conducting our analyses. Nonetheless, we conducted two sets of *post hoc* analyses aimed at uncovering evidence of bias. First, we examined whether confounding factors relating to age in our sample (i.e., generational and/or memory differences) led to systematic bias. We did find evidence of age differences in concussion reporting; however, these differences did not systematically bias our overall results. That is, we found no evidence that differences in reporting by individuals over 30, skewed the overall projections for concussion prevalence and number of concussions. Second, we conducted a *post hoc* analysis aimed at uncovering evidence of bias. Based on the logic that some select group respondents might systematically over-report the number of concussions and head impacts experienced, we examined the pattern of head impacts between those respondents reporting concussion versus those who did not report concussion. In this analysis, we found that although respondents reporting concussions did also report higher cumulative numbers of head impacts, the pattern of head impacts (games vs. practices) that they report is not significantly different from those of respondents that do not report concussions. Thus, this study’s limitations must be considered and addressed by future research. However, despite these limitations, our results highlight several differential patterns of vulnerability across the genders, across positions, and at different levels of play. These patterns cannot easily be explained by over-reporting or selection bias, but are more likely reflective of the specific risk factors of head injury at play in water polo.

We found that 534 or 36% of respondents reported sustaining at least one concussion while playing water polo and the average number of concussions that these respondents reported was 2.14. These numbers do suggest that exposure to water polo does carry a risk of concussion. Indeed, prevalence and the average number of reported concussions increase with the highest competition level reported and with the number of years played. Similar positive relationships were found between the cumulative number of blows to the head and years played. Our modeling of the data indicates that the highest competition level attained is the strongest predictor of concussion. Thus, it seems clear from these data that the amount of exposure to water polo is positively associated with concussion risk. Moreover, the concussion prevalence reported here is comparable to that observed in self-report studies in other sports. For instance, a 2014 NCAA survey of 20,000 current college athletes found that concussion prevalence in contact sports varied from 23.2% in men’s soccer to 29.2% in women’s ice hockey (10). Despite the differences between our methodology and this NCAA report,^3^ our findings are similar, and taken as a whole, we believe that our results warrant further investigation into the concussion risks in water polo.

Several of our survey questions suggest potential risk factors that could be targeted for future investigation. One such risk factor is field position. We found the prevalence of concussion to vary significantly as a function of field position (Table 1; Figure 1). Most notably, based on our data, goalies are at particular risk for concussion, with 47% of goalies reporting at least one concussion. The number of concussions that these goalies report was also significantly greater than for other positions. Importantly, this disproportionate risk in goalies was found for the majority of levels of play and therefore was not attributable simply to other factors such as years played. Additionally, we found that playing goalie significantly predicted the number of concussions. Interestingly, the patterns of head impacts that goalies report are quite different than those reported by other positions. For instance, goalies, unlike the other positions, report that most head impacts come during practice rather than during games. Goalies also report that the vast majority of head impacts come from the ball rather than from player contact. In contrast, other position players report that head impacts result from a combination of the ball and other from hits by other players.

These observed differences are wholly consistent with the nature of the goalie position. Goalies are the last line defensive player whose explicit objective is to prevent incoming offensive shots from reaching the goal (which is 3 m in length). As such, they are positioned at the ends of the pool facing mid-pool, with significant contact from the ball being propelled directly toward them. Goalies seek to deflect these shots with the front of their bodies. Since their lower bodies are submerged, water polo goalies predominately use their upper body to deflect shots, putting their heads at a disproportionate risk of being impacted. The exposure differences between games and practices noted above results from their spending a majority of practice time going through shot drills – without the presence of other defenders – where head impact exposure is heightened. Furthermore, in typical game situations, goalies rarely face as many shots as they face in practice. Thus, the unique nature of the goalie position and goalie practice may put this position at increased risk of concussion and head impact. Further empirical work will be needed to elaborate these factors. For instance, systematic investigation of head impacts during practice may be a fruitful step toward understanding why goalies report more concussions, why they have a higher projected lifetime prevalence of concussion, and how best to mitigate concussion risk within the context of the sport. We discuss more specific recommendations for goalies in the final section of this discussion.

As noted above, we found that reports of concussion varied across all levels of the sport and that projected lifetime prevalence and mean number of concussions were both associated with maximum level attained. This is consistent with the notion that increased exposure to water polo is a risk factor for concussion. One important question still remains, i.e., whether any particular levels of play carry disproportionate risk of concussion. In our results, we showed that two groups of players – those whose maximum level is professional (see Table S2 in Supplementary Material) and those whose maximum level is college – have the highest percentage of reported concussions. Based on amount of exposure and the physicality of professional sports, it is not surprising that there is high prevalence of concussion among professional water polo players. However, the high prevalence of concussion among maximum level college players is surprising, especially given that their average exposure, given by years of play, is typically lower than that of players of Masters’ club, Olympic, and Professional levels. From our data alone, it is hard to know what factors underlie the high rates of reported concussion in maximum level College players. One possibility is simply that there are more risk factors present during college. For instance college level water polo players, especially at the upper divisions, practice, scrimmage, and compete more often and for longer durations throughout the year. A second related possibility is that of attrition, i.e., a disproportionate number of college players who sustained concussions leave the sport after college. Given that the mean age of maximum level college players is lower than that of maximum level Masters’ Club players, another possibility is that College play is more physical. Regardless, future studies that more closely examine risk factors specific to College vs. Master’s Club and College vs. High School play are needed to better the high concussion rates in College.

We found significant gender differences in concussion consistent with a wealth of findings in the field (8, 9, 13, 14). Specifically, we found that a higher percentage of women than men reported sustaining at least one concussion as a result of water polo. This difference was present at almost all levels of the sport. By contrast, men reported, on average, numerically more concussions across levels and positions compared to women. This somewhat counterintuitive finding may suggest that men are more at risk for multiple concussions than women even though proportionately more women report concussions. Alternatively, this finding may reflect underreporting by men or differences in criterion between genders. It is of note that in our statistical modeling, gender was a significant predictor of sustaining at least one concussion, but not a significant predictor of the number of concussions reported. Thus taken as a whole, we hesitate to draw too strong or specific a conclusion regarding the exact nature of gender differences in our data. Gender differences are clearly an important factor for concussion that is being studied in the field, and given the high levels of participation by women in water polo (7); it is certainly a factor that needs to be considered in future studies on head injury in water polo.

Within the context of a self-report survey, it is not possible to test formally for clinical symptoms. Despite this limitation, it was our aim to gain a better understanding of the prevalence of PCS within our sample of water polo players. To assess PCS symptoms, we asked respondents if they experience “frequent headaches,” “difficulty sleeping,” and/or “irritability.” These are all common symptoms of PCS (4, 9). Our results do suggest an association between symptom reporting and concussion reporting. In particular, we found that the number of reported symptoms was a significant positive predictor of concussion prevalence and also number of concussions in respondents reporting concussion. This result, along with our findings on exposure and maximum level, support the notion that our questions about concussion have face validity.

### Proposed Recommendations

Our results speak to the clear need for systematic concussion reporting in water polo. In particular, reporting for individuals at the college level, who have among the highest prevalence of concussion, is especially vital. Second, our data strongly point to the possibility that goalies are at a disproportionate risk for concussion and head injury compared to other positions. Given that goalies report that most of their head impacts occur during practice, a means of reducing or mitigating harm during practice should be considered. In particular, it is possible that goalies wearing greater head protection during practice might be an effective way of mitigating concussion risk.

## Conclusion

These data provide an important and necessary first step in understanding the risks of concussion and head injury in the growing sport of water polo. We found that concussions are not uncommon in water polo, and that level of play, field position, and gender are critical factors in determining the risk of concussion in the sport. We recommend that concussion data in water polo be systematically reported, and that particular attention be paid to exposure risks of goalies during practice. The present study has clear limitations that must be considered. However, as the first epidemiological examination of head trauma in water polo, our study is successful in calling attention to the need and setting clear targets for systematic future laboratory and clinical studies on the specific mechanisms and risk factors of concussion in water polo.

## Ethics Statement

Institutional Review Board Human Subjects Committee, University of California, Irvine. No personally identifiable information was obtained, and the IRB determined that we did not need to obtain consent for survey participants. To participate in the study, participants clicked on a link to receive information on the study. Participants were informed about the aims of the study and that no personal identifying data would be collected and their individual results would be anonymous. Interested participants clicked next to take the survey.

## Author Contributions

RB, JW, JH, and SS designed the study, RB performed analyses, and RB, SS, JH, and JW wrote the paper.

## Conflict of Interest Statement

The authors do not have profession relationships with companies or manufacturers who might benefit from the present results.

## Footnotes

^1^Not all respondents reported gender.

^2^It should be noted that the opposite argument can be made for older individuals; memory and generational differences could lead to overreporting.

^3^The NCAA report did not state the definition of concussion that was used, it did not explain its participant selection procedure, nor did it explain its survey instrument.
