# Supplementary material for: The Epidemiology of Sports-Related Head Injury and Concussion in Water Polo
Source: Front Neurol. 2016 Jun 24;7:98. doi: 10.3389/fneur.2016.00098 (PMC4919321; doi:10.3389/fneur.2016.00098)
Supplement: Supplementary file 1 [file Table_1.DOCX]

Supplemental Table 1: Lifetime concussion prevalence and number of reported concussions

1. Lifetime Concussion Prevalence

| position | Attacker | | Utility | | 2mO | | 2mD | | Goalie | | **Total** | |
| --- | --- | --- | --- | --- | --- | --- | --- | --- | --- | --- | --- | --- |
| gender | Female | Male | Female | Male | Female | Male | Female | Male | Female | Male | **Female** | **Male** |
| All Levels | 52 (44.83%) | 61 (30.5%) | 57 (32.95%) | 45 (20.64%) | 38 (43.68%) | 47 (33.81%) | 55 (49.11%) | 50 (32.89%) | 55 (56.12%) | 66 (42.86%) | 257 (43.49%) | 272 (31.26%) |
|  |  |  |  |  |  |  |  |  |  |  |  |  |
| Age Group Club | 1 (7.69%) | 2 (5.88%) | 3 (17.65%) | 3 (6.98%) | 0 (n/a) | 3 (14.29%) | 0 (n/a) | 3 (17.65%) | 1 (33.33%) | 1 (6.67%) | 5 (9.43%) | 12 (9.09%) |
| High School | 22 (42.31%) | 22 (29.33%) | 25 (32.47%) | 16 (20.78%) | 12 (28.57%) | 14 (31.82%) | 23 (46%) | 15 (22.73%) | 21 (45.65%) | 18 (30%) | 103 (38.29%) | 85 (26.77%) |
| College | 11 (55%) | 10 (55.56%) | 12 (41.38%) | 5 (20%) | 7 (58.33%) | 7 (70%) | 19 (70.37%) | 7 (53.85%) | 16 (64%) | 7 (43.75%) | 65 (57.52%) | 36 (43.37%) |
| Masters Club | 16 (57.14%) | 20 (34.48%) | 9 (25.71%) | 15 (23.44%) | 16 (69.57%) | 19 (36.54%) | 11 (64.71%) | 18 (43.9%) | 11 (64.71%) | 33 (64.71%) | 63 (52.5%) | 105 (39.47%) |
| Olympic | 0 (n/a) | 4 (80%) | 2 (40%) | 0 (n/a) | 1 (33.33%) | 1 (16.67%) | 1 (50%) | 2 (50%) | 3 (75%) | 3 (50%) | 7 (46.67%) | 10 (47.83%) |
| Professional | 1 (100%) | 3 (30%) | 6 (60%) | 6 (85.71%) | 1 (100%) | 3 (50%) | 1 (25%) | 4 (40%) | 3 (100%) | 3 (60%) | 12 (63.16%) | 19 (50%) |

1. Number of reported concussions

| Position | Attacker | | Utility | | 2m Offense | | 2m Defense | | Goalie | | **Total** | |
| --- | --- | --- | --- | --- | --- | --- | --- | --- | --- | --- | --- | --- |
| Gender | Female | Male | Female | Male | Female | Male | Female | Male | Female | Male | **Female** | **Male** |
| All Levels | 1.9+/-0.2 | 2.1+/-0.3 | 2.1+/-0.2 | 2.4+/-0.5 | 2.1+/-0.2 | 3.2+/-1.1 | 2.0+/-0.2 | 2.5+/-0.5 | 2.3+/-0.2 | 3.4+/-0.7 | 2.0+/-0.1 | 2.8+/-0.3 |
|  |  |  |  |  |  |  |  |  |  |  |  |  |
| Age Group Club | 1** | 2+/-1 | 1** | 1.5+/-0.5 | n/a | 1.3+/-0.3 | n/a | 1+/-0.6 | n/a | 2** | 1** | 1.45+/-0.25 |
| High School | 1.8+/-0.3 | 1.3+/-0.1 | 1.6+/-0.2 | 1.4+/-0.2 | 1.9+/-0.2 | 1.4+/-0.1 | 1.6+/-0.2 | 3.2+/-1.6 | 1.5+/-0.1 | 1.3+/-0.2 | 2.4+/-0.2 | 2.3+/-0.5 |
| College | 1.9+/-0.3 | 1.4+/-0.2 | 2.8+/-0.7 | 2.4+/-0.52 | 2+/-0.4 | 4.3+/-2.0 | 2.5+/-0.3 | 1.7+/-0.4 | 2.5+/-0.4 | 2.1+/-0.4 | 1.7+/-0.1 | 1.8+/-0.3 |
| Masters Club | 2.0+/-0.4 | 2.4+/-0.1 | 2.2+/-0.5 | 3.3+/-1.3 | 2.1+/-0.3 | 4.6+/-2.6 | 2+/-0.4 | 2.6+/-0.5 | 3.2+/-0.4 | 5.0+/-1.3 | 2.3+/-0.2 | 3.8+/-0.7 |
| Olympic | n/a | 5.4+/-1.6 | 3+/-2 | n/a | 2** | 2** | 2** | 2+/-1 | 1.7+/-0.3 | 3.7+/-0.9 | 2.5+/-0.5 | 3.5+/-1.5 |
| Professional | 3** | 3.3+/-1.3 | 2.5+/-0.4 | 2.7+/-0.7 | 3** | 3.3+/-1.8 | 5** | 2.7+/-0.9 | 3.7+/-1.2 | 1.7+/-0.7 | 2.1+/-0.5 | 3.68+/-0.8 |
