# Supplementary material for: The Epidemiology of Sports-Related Head Injury and Concussion in Water Polo
Source: Front Neurol. 2016 Jun 24;7:98. doi: 10.3389/fneur.2016.00098 (PMC4919321; doi:10.3389/fneur.2016.00098)
Supplement: Supplementary file 2 [file Table_2.DOCX]

Supplemental Table2: Survey text

| 1.) What is your age? |  | |
| --- | --- | --- |
| 2.) What is your gender? | (M/F) | |
| 3.) How many years have you played water polo? |  | |
| 4.) Are you still playing water polo? | (Y/N) | |
| 5.) What levels of play have you participated in? | (Age Group Club, High School, College, Olympic, Professional, Masters Club) | |
| 6.) What position did/do you play the most? | (Attacker, Utility, 2m Offense, 2m Defense, Goalie) | |
| 7.) In a given practice, how often would you say you experience blows to the head? (please put a specific number) |  | |
| 8.) During a typical game, how often would you say you experience blows to the head? (please put a specific number) |  | |
| 9.) If you have experienced blows to the head, how is your head being impacted? (please answer each question) |  | |
| Back of the head | (None of the time, Some of the time, Most of the time, All of the time) | |
| Side of the head | (None of the time, Some of the time, Most of the time, All of the time) | |
| Front of the head | (None of the time, Some of the time, Most of the time, All of the time) | |
| Top of the head | (None of the time, Some of the time, Most of the time, All of the time) | |
| By the ball | (None of the time, Some of the time, Most of the time, All of the time) | |
| By another player | (None of the time, Some of the time, Most of the time, All of the time) | |
| 10.) In your experience, what level of play did you sustain the most serious blows to the head? | (Age Group Club, High School, College, Olympic, Professional, Masters Club) | |
| 11.) Did you ever have a concussion while playing water polo? DEFINITION of a CONCUSSION: A concussion is a blow to the head followed by a variety of symptoms that may include any of the following: headache, dizziness, loss of balance, blurred vision, "seeing stars", feeling in a fog or slowed down, memory problems, poor concentration, nausea, or throwing up. Getting "knocked out" or being unconscious does NOT always occur in concussion. If No: Proceed to Question 16. If Yes: Proceed to Question 12. | | (Y/N) |
| 12.) How many concussions do you think you have had while playing water polo? |  | |
| 13.) Did you ever lose consciousness or get "knocked out" as a result of a concussion? | (Y/N) | |
| 14.) Was there one or more seasons where you experienced multiple concussions? | (Y/N) | |
| If Yes, how many concussions did you experience? |  | |
| 15.) What level(s) of play did this occur? | (Age Group Club, High School, College, Olympic, Professional, Masters Club) | |
| 16.) Even if you don't think it resulted in a concussion, how many serious blows to the head have you had while playing water polo? |  | |
| 17.) Was there a year/season in which you had more than one serious blow to the head? | (Y/N) | |
| 18.) If yes to the previous question, how many seasons? |  | |
| 19.) Do you experience any of the following on a regular basis? | (Headaches, Problems Sleeping, Irritability) | |
| 20.) In what country have you played water polo the most (examples: USA, Australia, Hungary, etc.)? |  | |
