# Supplementary material for: The Epidemiology of Sports-Related Head Injury and Concussion in Water Polo
Source: Front Neurol. 2016 Jun 24;7:98. doi: 10.3389/fneur.2016.00098 (PMC4919321; doi:10.3389/fneur.2016.00098)
Supplement: Supplementary file 3 [file Table_3.DOCX]

Supplemental Table 3: Regression Models

1. Logistic Regression Model predicting lifetime concussion prevalence

|  | Odds | 95% Conf. Interval | Prob |
| --- | --- | --- | --- |
| Gender(Male) | 0.91 | [.85 0.96] | 2.30E-03 |
| Position(Goalie) | 1.12 | [1.02 1.23] | 1.13E-02 |
| Maximum Level | 1.05 | [1.03 1.08] | 1.00E-04 |

B) Multiple linear regression predicting the number of concussions reported

|  | beta | s.e. | t | Prob. | 95% Conf. Interval |
| --- | --- | --- | --- | --- | --- |
| Gender(Male) | -0.6195 | 0.261 | -2.373 | 0.018 | [-1.132 -0.107] |
| Position(Goalie) | -0.3282 | 0.442 | -0.743 | 0.458 | [-1.195 0.539] |
| Maximum Level | 0.2413 | 0.085 | 2.849 | 0.005 | [0.075 0.408] |
| Gender(Male)xPosition(Goalie) | 0.5485 | 0.62 | 0.885 | 0.376 | [-0.668 1.765] |
| Gender(Male)xMaximum Level | 0.1982 | 0.111 | 1.781 | 0.075 | [-0.02 0.417] |
| Maximum LevelxPosition(Goalie) | 0.3337 | 0.192 | 1.737 | 0.083 | [-0.044 0.711] |
| Gender(Male)xMaximum LevelxPosition(Goalie) | -0.0782 | 0.257 | -0.305 | 0.761 | [-0.583 0.426] |

C) Effect of adding symptoms to logistic regression model prediction lifetime concussion prevalence

|  | Odds | 95% Conf. Interval | Prob |
| --- | --- | --- | --- |
| Symptoms | 1.15 | [1.10 1.20] | 3.16E-10 |
