# Supplementary material for: The Epidemiology of Sports-Related Head Injury and Concussion in Water Polo
Source: Front Neurol. 2016 Jun 24;7:98. doi: 10.3389/fneur.2016.00098 (PMC4919321; doi:10.3389/fneur.2016.00098)
Supplement: Supplementary file 4 [file Table_4.DOCX]

Supplemental Table 4: Reported head impacts (mean with standard errors)

|  | Attacker | | Utility | | 2mO | | | 2mD | | Goalie | | **Total** | |
| --- | --- | --- | --- | --- | --- | --- | --- | --- | --- | --- | --- | --- | --- |
| gender | Female | Male | Female | Male | Female | | Male | Female | Male | Female | Male | **Female** | **Male** |
| All Levels | 5.03+/-0.56 | 6.37+/-0.89 | 5.53+/-1.31 | 3.75+/-0.44 | | 8.22+/-3.35 | 12.59+/-4.18 | 14.92+/-7.58 | 7.63+/-1.23 | 19.69+/-10.26 | 30.2+/-11.8 | 9.90+/-2.30 | 11.33+/-2.28 |
|  |  |  |  |  | |  |  |  |  |  |  |  |  |
| Age Group Club | 2.31+/-0.83 | 1.83+/-0.53 | 1.44+/-0.55 | 0.8+/-0.19 | | 1.4+/-0.6 | 2.8+/-0.77 | 1+/-0.37 | 1.5+/-0.68 | 4+/-3 | 0.94+/-0.34 | 1.63+/-0.33 | 1.48+/-0.22 |
| High School | 5.47+/-1.1 | 4.68+/-0.85 | 3.74+/-0.48 | 3.74+/-0.83 | | 4.74+/-0.7 | 6.36+/-2.2 | 5.74+/-0.74 | 4+/-1.12 | 7.24+/-1.27 | 5.23+/-0.8 | 5.18+/-0.38 | 4.63+/-0.49 |
| College | 4.92+/-0.9 | 6.09+/-1.79 | 4.8+/-0.74 | 2.83+/-0.58 | | 4.58+/-1.03 | 7.17+/-2.32 | 44.74+/-29.94 | 11.58+/-7.43 | 11.52+/-1.81 | 15.8+/-6.76 | 16.13+/-7.43 | 9.28+/-2.25 |
| Masters Club | 5.66+/-0.81 | 7.66+/-1.86 | 9.47+/-5.64 | 4.98+/-0.77 | | 18.91+/-12.83 | 17.57+/-10.09 | 6.29+/-1.74 | 11.01+/-2.08 | 11.71+/-2.27 | 57.87+/-30.87 | 10.26+/-2.98 | 18.85+/-6.18 |
| Olympic | 3.5** | 38.2+/-16.48 | 3.6+/-1.21 | 10** | | 6+/-4 | 16.2+/-6.85 | 3.75+/-1.25 | 17+/-11.1 | 263.5+/-245.73 | 206.5+/-161.75 | 78.21+/-7.43 | 72.68+/-45.16 |
| Professional | 5** | 11.45+/-5.09 | 15.6+/-9.48 | 15+/-5.75 | | 6** | 52.5+/-30.54 | 2.33+/-1.2 | 20.11+/-10.46 | 10.33+/-5.04 | 3.2+/-1.83 | 11.39+/-2.25 | 19.9+/-6.16 |
