# Supplementary material for: The Epidemiology of Sports-Related Head Injury and Concussion in Water Polo
Source: Front Neurol. 2016 Jun 24;7:98. doi: 10.3389/fneur.2016.00098 (PMC4919321; doi:10.3389/fneur.2016.00098)
Supplement: Supplementary file 5 [file Table_5.DOCX]

Supplemental Table 5: Symptoms

|  | Attacker | | Utility | | 2m Offense | | 2m Defense | | Goalie | | **Total** | |
| --- | --- | --- | --- | --- | --- | --- | --- | --- | --- | --- | --- | --- |
|  | Female | Male | Female | Male | Female | Male | Female | Male | Female | Male | **Female** | **Male** |
| no concussion | 0.39+/-0.08 | 0.29+/-0.05 | 0.66+/-0.08 | 0.21+/-0.04 | 0.61+/-0.14 | 0.23+/-0.05 | 0.46+/-0.11 | 0.21+/-0.05 | 0.7+/-0.15 | 0.31+/-0.07 | 0.57+/-0.05 | 0.24+/-0.02 |
| concussion | 1.04+/-0.14 | 0.77+/-0.14 | 1.18+/-0.15 | 0.36+/-0.08 | 1.34+/-0.19 | 0.53+/-0.12 | 1.35+/-0.13 | 0.88+/-0.14 | 1.22+/-0.16 | 0.83+/-0.14 | 1.22+/-0.07 | 0.71+/-0.06 |
